# Supplementary material for: Interventional therapy of extracranial arteriovenous malformations of the head and neck—A systematic review
Source: PLoS One. 2022 Jul 15;17(7):e0268809. doi: 10.1371/journal.pone.0268809 (PMC9286278; doi:10.1371/journal.pone.0268809)
Supplement: S1 Table — Complete list of terms used for database search. (DOCX) [file pone.0268809.s002.docx]

**S1 Table. PICO search terms.**

| POPULATION | INTERVENTION | OUTCOME |
| --- | --- | --- |
| „Arteriovenous Malformations“(M)  „Arteriovenous Fistula“(M)  Hemangioma (M)  “Lymphatic Abnormalities” (M)  “Vascular Malformations” (M)  “Port-Wine Stain” (M)  “lymphatic malformations”  “lymphatic malformation”  “capillary malformations”  “capillary malformation”  “venous malformations”  “venous malformation”  “vascular lesion”  “vascular lesions”  Lymphangioma, Cystic (M)  “Cystic Hygroma”  AND  “face” (M)  “mouth” (M)  “head”  neck  lip  face  facial  buccal  oral  mouth  gingiva  gingival  tongue  labial  labium  pharyngeal  oropharyngeal  pharynx  oropharynx  craniofacial  cranio-facial  “cranio-facial”  cervicofacial  cervico-facial  “cervico-facial”  cheek  chin  forehead | Sclerotherapy (M)  Percut*  Transcathet*  Emboliz*  Embolis*  Embolization, Therapeutic (M)  Chemoembolization, Therapeutic (M)  Manag*  Intervention  Interventions  Methods (M)  Procedures  Treat*  Therap*  Therapeutics (M)  Strategy | Progression  “Disease Progression” (M)  “Radiographic Progression”  Recurrence  Reinterven*  Esthetics (M)  “Quality of Life” (M) |

Complete list of terms used for database search.
