# Supplementary material for: Interventional therapy of extracranial arteriovenous malformations of the head and neck—A systematic review
Source: PLoS One. 2022 Jul 15;17(7):e0268809. doi: 10.1371/journal.pone.0268809 (PMC9286278; doi:10.1371/journal.pone.0268809)
Supplement: S2 Table — (DOCX) [file pone.0268809.s003.docx]

**S2 Table. Summary of the qualitative synthesis of methodology**

| Author  Diagnostic Test  THERAPY specified  outcome measurement  Previous treatment Reported  Follow-Up  Discription of symptoms  Discription of location  Specification of Complications | | | | | | | | |
| --- | --- | --- | --- | --- | --- | --- | --- | --- |
| Zou et al.(1) | **+** | **+** | **-** | **+** | - | **-** | **+** | **-** |
| Shobeirian et al.(2) | **+ +** | **+** | **-** | **+** | **+** | **+** | **+** | **+** |
| Kansy et al.(3) | **+ +** | **+** | **-** | **+** | **+ +** | **+** | **+** | **-** |
| Han et al.(4) | **+ +** | **+** | **+** | **+** | **+** | **+** | **+** | **+** |
| Chelliah et al.(5) | **+** | - | **+** | **+** | **+** | **+** | **+** | **+** |
| Wang et al.(6) | **+ +** | **+** | **+** | **+** | **+** | **+** | **+** | **+** |
| Meila et al.(7) | **+ +** | **-** | **+** | **+** | **+** | **+** | **+** | **+** |
| Su et al.(8) | **+ +** | **+** | **+** | - | **+ +** | **+** | **+** | **+** |
| Kim et al.(9) | **+** | **+** | **+** | **+** | **+ +** | **+** | **-** | **+** |
| Pekkola et al.(10) | **+ +** | **+** | **+** | **+** | **+** | **+** | **+** | **+** |
| Ermer et al.(11) | **+ +** | **+** | **-** | **+** | **+ +** | **+** | **+** | **+** |
| Pompa (2012) et al.(12) | **+** | **+** | **+** | **+** | **+** | **+** | **+** | **-** |
| Pompa (2011) et al.(13) | **+** | **+** | **+** | **+** | **+** | **+** | **+** | **-** |
| Richter et al.(14) | **+ +** | **+** | **-** | **+** | - | **+** | **+** | **-** |
| Fan et al.(15) | **+ +** | **+** | **+** | **+** | **+** | **+** | **+** | **+** |
| Gupta et al.(16) | **+ +** | **+** | **+** | **+** | **-** | **+** | **+** | **+** |
| Aslan et al.(17) | **+ +** | **+** | **+** | **+** | **+ +** | **+** | **+** | **+** |
| Chen et al.(18) | **+ +** | **+** | **+** | - | **+** | **+** | **+** | **+** |
| KajI et al.(19) | **+ +** | **+** | **-** | - | **-** | **-** | **+** | **+** |
| Saito et al.(20) | **+ +** | **+** | **-** | - | **+ +** | **+** | **+** | **+** |
| Hsiao et al.(21) | **+ +** | **+** | **+** | - | **+** | **+** | **+** | **+** |
| Zhao et al.(22) | **+ +** | **+** | **+** | - | **+ +** | **+** | **+** | **+** |
| Fujita et al.(23) | **+ +** | **+** | **-** | **+** | **+ +** | **+** | **+** | **+** |
| Gegenava et al.(24) | **+ +** | **+** | **-** | **+** | **+ +** | **+** | **+** | **-** |
| Ishimaru et al.(25) | **+ +** | **+** | **-** | **+** | **+ +** | **+** | **+** | **+** |
| Kitagawa et al.(26) | **+ +** | **+** | **+** | - | **+ +** | **+** | **-** | **+** |
| Dabus et al.(27) | **+ +** | **+** | **-** | **+** | **-** | **+** | **+** | **+** |
| Deng et al.(28) | **+ +** | **+** | **-** | - | **+** | **+** | **+** | **-** |
| Lemound et al.(29) | **+ +** | **+** | **-** | **+** | **+** | **+** | **+** | **+** |
| Spreafico et al.(30) | **+ +** | **+** | **-** | - | **+** | **+** | **+** | **+** |
| Manuel et al.(31) | **+ +** | **+** | **-** | - | **-** | **+** | **+** | **+** |
| Lai et al.(32) | **+ +** | **+** | **+** | **+** | **+ +** | **+** | **+** | **+** |
| DmytriW et al.(33) | **+ +** | **+** | **-** | **+** | **+ +** | **+** | **+** | **+** |
| Cariati et al.(34) | **++** | **-** | **-** | - | **+ +** | **+** | **+** | **-** |
| Bhandari et al.(35) | **+** | **+** | **+** | - | **+** | **+** | **+** | **+** |
| Churojana et al.(36) | **+ +** | **+** | **-** | **+** | **+ +** | **+** | **+** | **+** |
| Jafarian et al.(37) | **+ +** | **+** | **+** | - | **+ +** | **+** | **+** | **+** |
| Dixit et al.(38) | **+ +** | **-** | **-** | - | - | **+** | **+** | **+** |
| Son et al.(39) | **+ +** | **+** | **+** | - | - | **+** | **+** | **+** |
| Khambete et al.(40) | **+ +** | **+** | **-** | **+** | **+ +** | **+** | **+** | **+** |
| Ferrés-Amat et al.(41) | **+ +** | **+** | **+** | - | **+ +** | **+** | **+** | **-** |
| Atkinson et al.(42) | **+** | **-** | **-** | **+** | - | **+** | **+** | **+** |
| Yeh et al.(43) | **+ +** | **+** | **-** | - | **+ +** | **+** | **+** | **+** |
| PhillipS et al.(44) | **+ +** | **-** | **+** | **+** | **+ +** | **+** | **+** | **+** |
| Öztürk et al.(45) | **+ +** | **+** | **-** | **+** | + | **+** | **+** | **-** |
| Churojana et al.(46) | **-** | **+** | **-** | **+** | **-** | **+** | **+** | **+** |
| Oishi et al.(47) | **+ +** | **+** | **-** | **+** | **+** | **+** | **+** | **+** |
| Abdullah et al.(48) | **+ +** | **-** | **-** | - | **+** | **+** | **+** | **-** |
| Bhuyan et al.(49) | **+ +** | **+** | **-** | - | **+ +** | **+** | **+** | **+** |
| Wang et al.(50) | **+ +** | **+** | **+** | **+** | **-** | **+** | **+** | **+** |
| Zheng et al.(51) | **+ +** | **+** | **+** | **+** | **-** | **+** | **+** | **+** |
| Koshy et al.(52) | **+ +** | **+** | **-** | **+** | - | **+** | **+** | **+** |
| Hussain et al.(53) | **+ +** | **+** | **-** | **+** | - | **+** | **+** | **+** |
| Ou et al.(54) | **+ +** | **+** | **+** | **+** | - | **+** | **+** | **+** |
| Gupta et al.(55) | **+ +** | **+** | **-** | - | - | **+** | **+** | **+** |
| THIEX et al.(56) | **+ +** | **+** | **+** | **+** | **-** | **+** | **+** | **+** |

+ + = Gold Standard

+ = Item adequate

- = Item deemed inadequate
