# Supplementary material for: Interventional therapy of extracranial arteriovenous malformations of the head and neck—A systematic review
Source: PLoS One. 2022 Jul 15;17(7):e0268809. doi: 10.1371/journal.pone.0268809 (PMC9286278; doi:10.1371/journal.pone.0268809)
Supplement: S3 Table — (DOCX) [file pone.0268809.s004.docx]

**S3 Table. Overview of meta-analysed studies: Su et al.(12)**

| Su et al. 2015^12^ | Salvage treatment of hemorrhagic arteriovenous malformations in jaws |
| --- | --- |
| Clinical features and setting | The study enrolled patients who presented with a history of chronic intermittent interdental hemorrhage form AVM of the jaw between January 2012 and January 2014 to a university hospital (Shanghai Ninth People’s Hospital, China) |
| Participants | 32 patients were screened. 12 patients were eligible to be enrolled. The mean age of the patients was 16.5 years with a range from 9 to 22 years. Sex of the patients was not reported. No information on co-morbidity was provided. |
| Study design | Retrospective review of consecutive patients. |
| Diagnostic modalities | A CT scan done in all patients showed unilocular varix or ‘soap bubble’ AVM. Intraprocedural angiogram was performed. |
| Therapy applied | Emergency salvage of the bleed by interdental sling sutures or directly applied pressure with a gauze followed by transarterial absolute ethanol embolization in 10 patients with unilocular varix. In two patients with ‘soap bubble’-appearance varix, absolute ethanol was applied by direct puncture through the mental foramen. Deployment of coils in the venous pouch was performed before embolization. Post-interventional surgery was performed in patients with coils or with gingival dehiscence to remove granulated tissue or abscesses. |
| Results | 10 of 12 patients were cured. Two patients had partial remission and were waiting for additional sessions for residual AVM treatment of adjacent tissue. A single embolization session was usually sufficient. |
| Description of Outcome | Two radiologists evaluated the post-procedural CT scans and defined ‘cure’ as 100% devascularization of the nidus in comparison to the baseline CT and angiographical scan. Two oral and maxillofacial surgeons evaluated the clinical outcome. |
| Complications | Blood transfusions were given in 6 cases. There was no procedure-related mortality and no major complications. Minor complications included local skin necrosis, pyogenic granulomas at the puncture point, dehiscence of oral mucosa and loosening of teeth due to exposure, and localized necrosis of alveolar bone. |
| Description of Complications | Complications were classified as either major (death, permanent adverse sequelae, and requirement of major therapy) or minor (any non-permanent adverse sequelae, such as transient nerve injuries or spontaneously healing skin injury). |
| Follow-up | Follow-up ranged from 12 to 26 months (mean, 16.5 months) for all patients with periodic (1- to 3-month) evaluations on the basis of physical examination, panoramic radiographic views, and enhanced CT scans. |
