# Supplementary material for: Interventional therapy of extracranial arteriovenous malformations of the head and neck—A systematic review
Source: PLoS One. 2022 Jul 15;17(7):e0268809. doi: 10.1371/journal.pone.0268809 (PMC9286278; doi:10.1371/journal.pone.0268809)
Supplement: S4 Table — (DOCX) [file pone.0268809.s005.docx]

**S4 Table. Overview of meta-analysed studies: Kim et al.(13)**

| Kim et al 2015^13^ | Long-term results of ethanol sclerotherapy with or without adjunctive surgery for head and neck arteriovenous malformations |
| --- | --- |
| Clinical features and setting | Patients were managed by transarterial or direct-puncture ethanol sclerotherapy with or without combined surgical removal between April 1997 and December 2013 at the vascular malformation clinic of the Sungkyunkwan University School of Medicine in Seoul, South Korea. |
| Participants | 120 patients with head and neck AVM were screened. 49 of them, who did not receive treatment as well as three who were managed by surgical intervention alone and those who did not have one year follow-up were excluded. 45 patients were subjected to endovascular treatment and included in the analysis. |
| Study design | Retrospective chart review of consecutive patients |
| Diagnostic modalities | After physical examination, all patients underwent baseline CTA, MRI, or a combination of the two modalities. The angioarchitecture of the lesions was reviewed and classified according to the Schobinger staging system ^90^. |
| Therapy applied | After superselective angiography of the affected branches of the external carotid artery, direct-puncture sclerotherapy was performed under fluoroscopic guidance. Ethanol at various concentrations from 50 to 99% was used. The total amount of ethanol did not exceed 1ml/kg bodyweight. |
| Results | Complete angiographic eradication was seen in 17.8% (8/45 patients). All of them had complete remission of the clinical symptoms. Partial resolution was achieved in 34 patients, and no response was observed in three. |
| Description of Outcome | Posttreatment assessment was performed by two interventional radiologists, who compared the baseline images with the final ones. Complete resolution or cure was defined as devascularization of more than 99%, and partial resolution was defined as 50-99% devascularization. An overall favorable outcome was observed in 77.8% (n=34) of patients. |
| Complications | 28 patients developed 34 minor complications such as necrosis (n=15), skin bullae (n=12), skin discoloration (n=3), muscular ulcer (n=2), bleeding (n=1) and transient nerve palsy (n=1). Other intervention related clinical findings were increase of pulmonary pressure (n=6), hypesthesia (n=2), arrhythmias (n=1), numbness (n=1) and dizziness (n=1). Major complications were seen in five patients with skin necrosis requiring graft transplantation in three. One patient suffered from increased orbital pressure that was treated with surgical decompression. One suffered from blindness, weakness and dysarthria due to cortical infarction. |
| Description of Complications | Major complications were death, permanent adverse sequelae, the need for major therapy, or prolonged hospitalization (>48 h). Minor complications were no permanent adverse sequelae, such as skin discoloration, mucosal ulcer, bleeding, transient nerve injuries, or skin necrosis that can healed spontaneously. Skin necrosis that required a skin graft was considered a major complication. |
| Follow-up | Mean follow-up period was 56.6 months (range, 13–144 months) |
