# Supplementary material for: Interventional therapy of extracranial arteriovenous malformations of the head and neck—A systematic review
Source: PLoS One. 2022 Jul 15;17(7):e0268809. doi: 10.1371/journal.pone.0268809 (PMC9286278; doi:10.1371/journal.pone.0268809)
Supplement: S5 Table — (DOCX) [file pone.0268809.s006.docx]

**S5 Table. Comprehensive overview of reviewed articles not eligible for meta-analysis**

| **Author** | **Study design** | **Patient population** | **Diagnostic measures** | **Therapy** | **Results** | **Follow-Up** |
| --- | --- | --- | --- | --- | --- | --- |
| Zou, Y. ^33^ | Retrospective chart review | Total of 446 patients with n=18 treated, sex and age not specified for the treatment group | CT or MRI | Bleomycin | n=8 partial remission, n=7 no change, n=3 recurrence clinically | mean of whole cohort 750 days |
| Shobeirian ^23^ | Case report | 1 male, 70 years old | Doppler-US, CT, CTA | Transarterial embosphere | no recurrence | 180 days |
| Kansy ^21^ | Monocentric retrospective analysis | n=13, 6 males, 7 females, mean age 39 years (15-67) | Angiogram | Direct puncture Onyx, Transarterial Embosphere or NBCA | n=11 complete, n=2 partial measured by Wu et al. ^91^ | mean 804 days (360-1800) |
| Han ^39^ | Retrospective chart review | n=15, 8 males, 7 females, mean age 24 years (9-39) | Selective Angiogram | Transarterial coil deployment | n=8 100% devascularization, n=7 partial | mean 510 days (30-1440) |
| Chelliah ^15^ | Case series | n=6, 4 males, 2 females, mean age 24,5 years (9-44) | MRI | Sirolimus and embolization (agent not specified) | n=2 notable reduction and no recurrence, n=4 partial reduction | mean 570 days (180-1200) |
| Wang ^50^ | Retrospective chart review | n=18, 12 males, 6 females, mean age 20,9 years (10-35) | CT, Angiogram, DSA | Transarterial ethanol and coils | n=9 100% devascularization, n=9 partial remission | mean 471 days (240-780) |
| Meila ^14^ | Retrospective chart review | n=14, sex and age not specified | DSA, MRI | Embolization (agents not specified) | n=2 100% devascularization, n=4 > 90% devascularization, 8 partial | mean 2730 days (200-4860) |
| Pekkola ^52^ | Retrospective chart review | n=19, 7 males, 12 females, mean age 24,4 years (6-47) | DSA in all patients, MRI in some | Transarterial NBCA or Onyx or ethanol with coils or balloons | n=11 complete shunt eradication and resolved symptoms, n=8 partial, n=1 no change | mean 290 days (150-900) |
| Ermer ^24^ | Case report | 1 male, 30 years old | MRI, DSA | Ethibloc with surgery and ethanol with surgery | Stable condition without further embolization | 1080 days |
| Pompa ^31^ | Case series | n=23, 14 males, 9 females, mean age 29,2 years (6-42) | MRI or CT, or Angiography | Transarterial PVA or coils | n=8 with 90% devascularization, n=15 between 80%-40% | mean 780 days (180-1800) |
| Pompa ^60^ | Retrospective chart review | n=20, 13 males, 7 females, mean age 23,4 years (6-42) | MRI or CT, or Angiography | Transarterial PVA or Gelfoam or coils | n=7 very good, n=10 good, n=2 sufficient, each item not defined | mean 780 days (180-1800) |
| Richter ^26^ | Retrospective chart review | n=10, 6 males, 4 females, mean age 33 years (13-46) | MRI, Arteriogram | Transarterial PVA and surgery | n=8 complete, n=2 partial by interview and Wu et al. ^91^ | mean 792 days |
| Fan ^26^ | Retrospective chart review | n=8, 4 males, 4 females, mean age 23,5 years (14-43) | CT, DSA | Transarterial ethanol with or without coils | n=6 100% devascularization, n=2 partial remission | mean 126 days (30-240) |
| Gupta ^64^ | Retrospective chart review | n=15, 9 males, 6 females, age range 7-59 years | CT, Angiogram | Direct-puncture NBCA or transarterial PVA with Gelfoam | n=11 complete disappearance in angiogram, n=4 >90% devascularization | Range 180 days -3960 days |
| Aslan ^65^ | Case report | 1 female, 33 years old | MRI, CT, selective angiogram | Transarterial PVA and surgery | MRI showed no residual lesion | 1080 days |
| Chen ^66^ | Retrospective chart review | n=13, 10 males, 3 females, mean age 9,1 years (4-13) | DSA, 3D-CTA | Transarterial Bleomycin or OK432 and surgery | n=7 >90% devascularization, n=5 mostly involuted, n=1 partially involuted | mean 405 days (180-660) |
| Kaji ^27^ | Retrospective chart review | n=23, sex and age not specified | Scintigraphy, MRI | Transarterial NBCA and Ethanolamine Oleate | n=6 definite reduction obtained, n=9 slight, n=7 no change or worse | Range 90 days to 450 days |
| Saito ^67^ | Case report series | 2 females, 43 and 45 years old | MRA | Percutaneous Polidocanol | n=2 cosmetically complete resolved | mean 900 days (720-1080) |
| Hsiao ^22^ | Case report | 1 male, 42 years olds | CT, Angiogram | Transarterial Onyx | Retention with the vascular lesion | 30 days |
| Zhao ^58^ | Retrospective chart review | n=13, 8 males, 5 females, mean age 28 years (16-46) | 3D-CTA | Transarterial PVA and Fibrin Glue and OK432 and Bleomycin | n=3 >90% reduction, n=10 nearly complete or partial | mean 822 days (360-1740) |
| Fujita ^25^ | Case report | 1 female, 30 years old | MRI, CTA, Angiography | Transarterial NBCA or ethanol | Reduction in size of lesions, no further hemorrhage | 930 days |
| Gegenava ^68^ | Case report | 1 male, 36 years old | CT, selective angiograph | Transarterial Embosphere and PVA and Aethoxysclerol Onyx | No report of recurrence of bleeding by telephone. | 720 days |
| Ishimaru ^69^ | Retrospective chart review | n=5, 1 male, 4 females, mean age 38 years (20-50) | Angiograph, | Transarterial NBCA and coils or Polymere microsphere beads or surgery | All with subside symptoms | mean 1152 days (1080-1260) |
| Dabus ^70^ | Case series | n=18 with 31 lesions, 6 males, 12 females, mean age 45 years (4-66) | Angiograph, | Onyx, ethanol, NBCA and coils used via different routes | 14/31 lesions completely resolved, 9/31 partial, 8/31 without change (no definitions) | mean 300 days |
| Deng ^71^ | Retrospective chart review | n=16, 11 males, 5 females, mean age 32,4 years (18-49) | CT, DSA | Transarterial PVA, coils or NBCA | All clinically disappeared with most of the radiolucency disappeared | mean 1140 days (210-1890) |
| Lemound ^72^ | Case series | 2 females, 8 and 15 years old | CT, Angiogram | Transarterial Bead Blocks and surgery | Uneventful postoperative course | mean 345 days (210-510) |
| Spreafico ^73^ | Case report | 1 female, 23 years old | MRI, MRA | Transarterial coils with STS and surgery | Almost complete reabsorption | 180 days |
| Manuel ^74^ | Case report | 1 male, 35 years old | MRI, Angiogram | Transarterial NBCA and PVA and surgery | Still residual lesions in imaging | Not specified |
| Lai ^75^ | Case report | 1 female, 19 years old | MRI, DSA | Transarterial PVA and NBCA with Lipiodol | No recurrence | 1080 days |
| Dmytriw ^56^ | Case report | 1 male, 32 years old | CTA, 3D-CTA | Transarterial NBCA, Ethanol embolization, bevacizumab, Bleomycin, Onyx and surgery | Patient died after multiple embolizations and surgical interventions | 5 years |
| Cariati ^16^ | Case report | 1 male, 11 years old | CT, selective angiogram, MRI | Transarterial Embolization (no agent specified) | Significant reduction in control angiogram and no recurrence | 18 months |
| Bhandari ^61^ | Retrospective chart review | n=20, 8 males, 12 females, age range 4 to 56 | Color Doppler, MRA | PVA, NBCA, Gelatine Sponge particles and surgery | n=14 lesions completely devascularized, n=5 effectively | mean 15,2 months (5 months – 3.5 years) |
| Churojana ^76^ | Retrospective chart review | n=5, 3 males, 2 females, mean age 12,4 | CT, angiography | NBCA (transarterial or transosseous), transarterial glue and lipiodol | n=4 patients complete cure without recurrence, one good control of bleeding | mean 6.6 years (1 – 19 years) |
| Jafarian ^77^ | Case report | 1 female, 18 years old | Selective angiogram | intralesional STS | Angiogram showed significant decrease, no recurrence | 2 years |
| Dixit ^17^ | Case report | One male, 21 years old | MRA, 3D-TOF | Sclerotherapy (No agent specified) | Uneventful post-interventional period | Not specified |
| Son ^78^ | Case report | One male, 31 years old | CT, MRI, Angiography | Transarterial Onyx | Uneventful recovery | Lost to follow-up |
| Khambete ^79^ | Case report | One female, 30 years old | US, Angiography | Transarterial PVA and surgery | Significant reduction of clinical symptoms | One year |
| Ferrés-Amat ^80^ | Case report | One female, 9 years old | CT, MRA, Angiography | Transarterial NBCA and Lipiodol, Direct-puncture Ethanol of zein | Imaging showed control of lesion with ablation of all branches | 8 years follow-up |
| Atkinson ^18^ | Case report | One female, 11 years old | CT | Embolization (No agent specified), surgery | Not specified | Not specified |
| Yeh ^81^ | Case report | One female, 10 years old | CT, Angiogram | Transarterial NBCA | Recurrent bleeding with additional embolization. No more recurrence | Two years |
| Phillips ^19^ | Retrospective chart review | n=26, 9 males, 17 females, mean age 8 years | Angiogram or MRI and biopsy | Embolization (no agent specified) | n=2 lesions obliterated, 1/3 with reduced AVM, n=2 with increased lesion | Mean 7.5 years, minimum 5 years |
| Öztürk ^82^ | Case report | One male, 12 years old | CT, Angiography | Transarterial NBCA with Lipiodol and surgery | Nidus was totally embolized | 4 months |
| Churojana ^83^ | Retrospective chart review | n=33 with 19 AVM, 15 male, 18 female, mean age 28 years (8-51) | Clinical examination, arteriogram only if diagnosis not obvious | Transarterial NBCA | n=12 cured (lesions clinically undetecdable), 21 satisfactory | Not specified |
| Oishi ^84^ | Case report | One male, 21 years old | Angiography | Transarterial ethanol and Eudragit-E | Angiogram showed reduction/nearly total disappearance of AVM | One week |
| Abdullah ^20^ | Case report | One female, 15 years old | CT, MRI, MRA | Embolization (no agent specified) with surgery | Patient recovered well | Not specified |
| Bhuyan ^85^ | Case report | One female, 4 years old | CT, CTA | Transarterial PVA | Patient with stable health status and no bleeding | One year |
| Wang ^59^ | Case report | One male, 15 years old | Angiography | Transarterial PVA and surgery | Complete devascularization of AVM on angiogram | 6 months |
| Zheng ^62^ | Retrospective chart review | n=17, 11 males, 6 females, mean age 25,4 years (3-47) | Angiography | Transarterial or direct-puncture ethanol injection | n=3 with 100% devascularization, n=5 with 76-99%, n=6 with 50-75%, n=3 with <50% | Mean 6,5 months (range 1-16) |
| Koshy ^86^ | Case report | One male, 11 years old | CT, Doppler, MRI, DSA | Transarterial NBCA and direct intravenous injection | MRI showed thrombosis within the vascular space | Ongoing |
| Hussain ^87^ | Case report | One male, 10 days old | US, CTA, DSA | Transarterial PVA and coils with surgery | No development of complications and in stable condition | Not specified |
| Ou ^88^ | Case series | n=4, 2 males, 2 females, mean age 51,5 years (30-66) | Angiogram | Direct-puncture NBCA with Lipiodol with surgery | All 4 lesions completely eradicated on angiogram | Not specified |
| Gupta ^57^ | Case report | One male, 24 years old | MRI, MRA | Coil embolization and surgery | Ligation of all tortuous vessels and whole resection of ear | Not specified |
| Thiex ^89^ | Retrospective chart review | n=18, 10 males, 8 females, mean age 30,1 years (6-65) | Angiography | Transarterial Onyx with surgery in 5 | Significant reduction of AV-shunt in 8, 1 recurrence | 6 months |
