# Supplementary material for: Interventional therapy of extracranial arteriovenous malformations of the head and neck—A systematic review
Source: PLoS One. 2022 Jul 15;17(7):e0268809. doi: 10.1371/journal.pone.0268809 (PMC9286278; doi:10.1371/journal.pone.0268809)
Supplement: S1 Appendix — (DOCX) [file pone.0268809.s007.docx]

**S6 Appendix. Complete PubMed search string.**

(((((((((((((((((("arteriovenous malformations"[MeSH Terms]) OR "arteriovenous fistula"[MeSH Terms]) OR Hemangioma) OR "lymphatic abnormalities"[MeSH Terms]) OR "vascular malformations"[MeSH Terms]) OR ("port-wine stain"[MeSH Terms] OR ("port-wine"[All Fields] OR "port-wine stain"[All Fields] OR "port wine stain"[All Fields])) OR "lymphatic malformations") OR "lymphatic malformation") OR "capillary malformations") OR "capillary malformation") OR "venous malformations") OR "venous malformation") OR "vascular lesion") OR "vascular lesions") OR ("lymphangioma, cystic"[MeSH Terms] OR ("lymphangioma"[All Fields] AND "cystic"[All Fields]) OR "cystic lymphangioma"[All Fields] OR ("lymphangioma"[All Fields] AND "cystic"[All Fields]) OR "lymphangioma, cystic"[All Fields]))) AND ((((((((((((((((((((((((((("face"[MeSH Terms] OR "face"[All Fields])) OR ("mouth"[MeSH Terms] OR "mouth"[All Fields])) OR neck) OR lip) OR face) OR facial) OR buccal) OR oral) OR gingiva) OR gingival) OR tongue) OR labial) OR labium) OR pharyngeal) OR oropharyngeal) OR pharynx) OR oropharynx) OR craniofacial) OR cranio-facial) OR "cranio-facial") OR cervicofacial) OR cervico-facial) OR "cervico-facial") OR cheek) OR chin) OR forehead))) AND ((((((((((((((((Sclerotherapy) OR (percute[All Fields] OR percuteaneous[All Fields] OR percuteanously[All Fields] OR percutem[All Fields] OR percutemincola[All Fields] OR percutenaous[All Fields] OR percuteneous[All Fields] OR percuteneousnephrostomy[All Fields] OR percuteneus[All Fields] OR percutenous[All Fields] OR percutenuous[All Fields])) OR (transcathetar[All Fields] OR transcathete[All Fields] OR transcathetel[All Fields] OR transcatheter[All Fields] OR transcatheteral[All Fields] OR transcatheterally[All Fields] OR transcatheteraortic[All Fields] OR transcatheterarterial[All Fields] OR transcatheterasd[All Fields] OR transcathetereal[All Fields] OR transcatheterembolization[All Fields] OR transcatheteric[All Fields] OR transcatheterically[All Fields] OR transcatheterimplantation[All Fields] OR transcatheterisation[All Fields] OR transcatheterization[All Fields] OR transcatheterly[All Fields] OR transcatheters[All Fields] OR transcatheteter[All Fields] OR transcathether[All Fields] OR transcathetheral[All Fields] OR transcathetheter[All Fields] OR transcathetor[All Fields] OR transcathetral[All Fields])) OR (embolizable[All Fields] OR embolization'[All Fields] OR embolization's[All Fields] OR embolization,[All Fields] OR embolizational[All Fields] OR ("embolization, therapeutic"[MeSH Terms] OR ("embolization"[All Fields] AND "therapeutic"[All Fields]) OR "therapeutic embolization"[All Fields] OR "embolizations"[All Fields]) OR embolizaton[All Fields] OR embolize[All Fields] OR embolized[All Fields] OR embolizer[All Fields] OR embolizers[All Fields] OR embolizes[All Fields] OR emboliziation[All Fields] OR embolizing[All Fields] OR embolizm[All Fields] OR embolizma[All Fields])) OR (embolis[All Fields] OR embolisa[All Fields] OR embolisable[All Fields] OR embolisace[All Fields] OR embolism's[All Fields] OR embolism,[All Fields] OR embolismal[All Fields] OR embolisme[All Fields] OR embolismic[All Fields] OR embolismimpact[All Fields] OR embolisms[All Fields] OR embolismus[All Fields])) OR ("chemoembolization, therapeutic"[MeSH Terms] OR ("chemoembolization"[All Fields] AND "therapeutic"[All Fields]) OR "therapeutic chemoembolization"[All Fields] OR ("chemoembolization"[All Fields] AND "therapeutic"[All Fields]) OR "chemoembolization, therapeutic"[All Fields])) OR management) OR managing) OR Interventions[All Fields]) OR ("methods"[MeSH Terms] OR "methods"[All Fields] OR "intervention"[All Fields])) OR ("methods"[MeSH Terms] OR "methods"[All Fields] OR "procedure"[All Fields])) OR treatment) OR treating) OR treat) OR ("therapy"[Subheading] OR "therapy"[All Fields] OR "therapeutics"[MeSH Terms] OR "therapeutics"[All Fields])) OR strategy[All Fields])) AND ((((((((Progression) OR ("disease progression"[MeSH Terms] OR ("disease"[All Fields] AND "progression"[All Fields]) OR "disease progression"[All Fields] OR "progression"[All Fields])) OR "Radiographic Progression") OR ("recurrence"[MeSH Terms] OR "recurrence"[All Fields])) OR reintervention) OR reinterventions) OR ("esthetics"[MeSH Terms] OR "esthetics"[All Fields])) OR ("quality of life"[MeSH Terms] OR ("quality"[All Fields] AND "life"[All Fields]) OR "quality of life"[All Fields]))
