# Supplementary material for: Interventional therapy of extracranial arteriovenous malformations of the head and neck—A systematic review
Source: PLoS One. 2022 Jul 15;17(7):e0268809. doi: 10.1371/journal.pone.0268809 (PMC9286278; doi:10.1371/journal.pone.0268809)
Supplement: S2 Appendix — (DOCX) [file pone.0268809.s008.docx]

**S7 Appendix Complete list of excluded studies.**

| **Title** | **Author** | **Year** |
| --- | --- | --- |
| Intralesional bleomycin injection in management of low flow vascular malformations in children | Mohan, A. T. | 2015 |
| Haemangiomas and venous malformations of the head and neck: A retrospective analysis of endovascular management in 358 patients | Sachin, K. | 2013 |
| Sclerotherapy for Eyelid and Anterior Orbital Venous-Lymphatic Malformation | Razavi, ME | 2019 |
| A single-center experience in the management of head and neck lymphangiomas | Aluffi Valletti, P. | 2020 |
| Lymphatic Malformation Architecture: Implications for Treatment With OK-432 | Malic, C. C. | 2017 |
| Bleomycin sclerotherapy for lymphatic malformation after unsuccessful surgical excision: case report | Vlahovic, A. | 2015 |
| Intralesional bleomycin injection in management of low flow vascular malformations in children | Mohan, A. T. | 2015 |
| Efficacy of OK-432 sclerotherapy in treatment of lymphatic malformations: long-term follow-up results | Weitz-Tuoretmaa, A. | 2014 |
| OK432 versus doxycycline for treatment of macrocystic lymphatic malformations | Motz, K. M. | 2014 |
| Management of the low-flow head and neck vascular malformations in children: the sclerotherapy protocol | Leung, M. | 2014 |
| Haemangiomas and venous malformations of the head and neck: A retrospective analysis of endovascular management in 358 patients | Sachin, K. | 2013 |
| Pingyangmycin with triamcinolone acetonide effective for treatment of lymphatic malformations in the oral and maxillofacial region | Luo, Q. F. | 2013 |
| New treatment for cystic lymphangiomas of the face and neck: cyst wall rupture and cyst aspiration combined with sclerotherapy | Mitsukawa, N. | 2012 |
| Bleomycin A5 sclerotherapy for cervicofacial lymphatic malformations | Yang, Y. | 2012 |
| Treatment of lymphangioma circumscriptum with sclerotherapy: an ignored effective remedy | AlGhamdi, K. M. | 2011 |
| Sclerotherapy for cervical cystic lymphatic malformations in children. Our experience with computed tomography-guided 98% sterile ethanol insertion and a review of the literature | Impellizzeri, P. | 2010 |
| Efficacy and safety of OK-432 immunotherapy of lymphatic malformations | Smith, M. C. | 2009 |
| Percutaneous treatment of lymphatic malformations | Shiels, W. E., 2nd | 2009 |
| [Treatment of lymphangiomas with picibanil in the first year of life] | Subotic, U. | 2008 |
| Doxycycline sclerotherapy as primary treatment of head and neck lymphatic malformations in children | Nehra, D. | 2008 |
| Noninterventional treatment of selected head and neck lymphatic malformations | Dasgupta, R. | 2008 |
| Percutaneous sclerotherapy of lymphatic malformations with doxycycline | Burrows, P. E. | 2008 |
| OK432 (picibanil) efficacy in an adult with cystic cervical lymphangioma. A case report | Alonso, J. | 2005 |
| OK-432 and lymphatic malformations in children: the Starship Children's Hospital experience | Wheeler, J. S. | 2004 |
| Management of orbital lymphangioma using intralesional injection of OK-432 | Suzuki Y. | 2000 |
| Safety and Efficacy of Surgery Combined With Bleomycin Irrigation for Complex Cervical-Facial Lymphatic Malformations of Children | Wang, Y. | 2020 |
| Percutaneous Lauromacrogol Foam Sclerotherapy for the Treatment of Acute Airway Compression Caused by Lymphatic Malformations in Infants | Wang, L. | 2018 |
| Image-Guided Percutaneous Bleomycin and Bevacizumab Sclerotherapy of Orbital Lymphatic Malformations in Children | Abdelaziz, O. | 2018 |
| Sclerotherapy with picibanil (OK-432) for congenital lymphatic malformation in the head and neck | Sung, M. W. | 2001 |
| Sclerotherapy for congenital lesions in the head and neck | Kim, K. H. | 2004 |
| Percutaneous sclerotherapy for lymphatic malformations: a retrospective analysis of patient-evaluated improvement | Alomari, A. I. | 2006 |
| Treatment of Cystic Lymphatic Vascular Malformations with OK-432 Sclerotherapy | Peters, D. A. | 2006 |
| Doxycycline sclerotherapy as the primary treatment for head and neck lymphatic malformations | Cordes, B. M. | 2007 |
| Sclerotherapy of Microcystic Lymphatic Malformations in Oral and Facial Regions | Bai, Y. | 2009 |
| Multimodality treatment of pediatric lymphatic malformations of the head and neck using surgery and sclerotherapy | Boardman, S. J. | 2010 |
| Percutaneous sclerotherapy of massive macrocystic lymphatic malformations of the face and neck using fibrin glue with OK-432 and bleomycin | Chen, W. L. | 2010 |
| Lymphatic malformations: clinical course and management in 64 cases | Hogeling, M. | 2011 |
| Percutaneous drainage and ablation as first line therapy for macrocystic and microcystic orbital lymphatic malformations | Hill, R. H. | 2012 |
| Doxycycline sclerotherapy for pediatric head and neck macrocystic lymphatic malformations: a case series and review of the literature | Jamal, N. | 2012 |
| Doxycycline sclerotherapy in children with lymphatic malformations: outcomes, complications and clinical efficacy | Shergill, A. | 2012 |
| Percutaneous sclerotherapy with ethanolamine oleate for lymphatic malformations of the head and neck | Alexander, M. D. | 2014 |
| Safety and efficacy of bleomycin sclerotherapy for microcystic lymphatic malformation | Chaudry, G. | 2014 |
| OK-432 sclerotherapy of lymphatic malformation in the head and neck: factors related to outcome | Kim, D. W. | 2014 |
| Efficacy of doxycycline and sodium tetradecyl sulfate sclerotherapy in pediatric head and neck lymphatic malformations | Farnoosh, S. | 2015 |
| Patients with lymphatic malformations who receive the immunostimulant OK-432 experience excellent long-term outcomes | Ghaffarpour, N. | 2015 |
| Total clinical regression of an orbital macrocystic lymphatic malformation following intralesional sodium tetradecyl sulphate injection | Kiratli, H. | 2015 |
| Sclerotherapy treatment of orbital lymphatic malformations: a large single-centre experience | Barnacle, A. M. | 2016 |
| Cervicofacial Lymphatic Malformations: A Retrospective Review of 40 Cases | Cho, B. C. | 2016 |
| Doxycycline Sclerotherapy Is Superior in the Treatment of Pediatric Lymphatic Malformations | Thomas, D. M. | 2016 |
| Treatment of deep-seated facial microcystic lymphatic malformations with intralesional injection of pingyangmycin | Wu, H. W. | 2016 |
| Large facial lymphatic malformation treatment using sclerosing agent followed by surgical resection: clinical and pathology report | Laviv, A. | 2017 |
| Experience of sclerotherapy and embolosclerotherapy using ethanolamine oleate for vascular malformations of the head and neck | Kaji, N. | 2009 |
| Percutaneous sclerotherapy for lymphangioma and soft-tissue venous malformation located in the maxillofacial region in children and young adults | Graziuso, S. | 2005 |
| Sclerotherapy for vascular malformations: complications and a review of techniques to avoid them | Odeyinde, S. O. | 2013 |
| Percutaneous sclerotherapy with gelified ethanol of low-flow vascular malformations of the head and neck region: preliminary results | Ierardi, A. M. | 2019 |
| Is Multiple Session of Intralesional Bleomycin Mandatory for Complete Resolution of Macrocystic Lymphatic Malformation? | Upadhyaya, V. | 2018 |
| Use of Percutaneous Bleomycin Sclerotherapy for Orbital Lymphatic Malformations | Hanif, A. | 2019 |
| Aqueous Intralesional Bleomycin Sclerotherapy in Lymphatic Malformation: Our Experience With Children and Adult | Bhatnagar, A. | 2017 |
| Percutaneous ultrasound-guided sclerotherapy with polidocanol microfoam for lymphatic malformations | Yamaki, T. | 2017 |
| Percutaneous sclerotherapy of pediatric lymphatic malformations: experience and outcomes according to the agent used | Gallego Herrero C | 2017 |
| Single-stage embolization with n-butyl cyanoacrylate and surgical resection of venous malformations | Polites, S. F. | 2020 |
| Bleomycin sclerotherapy for eyelid venous malformations as an alternative to surgery or laser therapy | Shigematsu, T. | 2019 |
| Venous malformations of the head and neck: A retrospective review of 82 cases | Park, H. | 2019 |
| Sclerotherapy as an esthetic indication in oral vascular malformations: a case series | Manzano, B. R. | 2019 |
| Efficacy of Percutaneous Sclerotherapy in Low Flow Venous Malformations - A Single Center Series | Ahmad, S. | 2019 |
| Quality of life after endovascular sclerotherapy of low-flow venous malformations: the efficacy of polidocanol compared with ethanol | Weitz-Tuoretmaa, A. | 2018 |
| Effectiveness and safety of foam sclerotherapy with 5% ethanolamine oleate in the treatment of low-flow venous malformations in the head and neck region: a case series | Ribeiro, M. C. | 2018 |
| Effectiveness and Safety of Sclerotherapy for Treatment of Low-Flow Vascular Malformations of the Oropharyngeal Region | Bourgouin, P. | 2018 |
| Ethanol sclerotherapy for venous malformation | Steiner, F. | 2017 |
| Clinical outcome and predictors of treatment response in foam sodium tetradecyl sulfate sclerotherapy of venous malformations | Park, H. S. | 2016 |
| Sclerotherapy using 1% sodium tetradecyl sulfate to treat a vascular malformation: a report of two cases | Min, H. G. | 2015 |
| Sclerotherapy using 1% sodium tetradecyl sulfate to treat a vascular malformation: a report of two cases | Min, H. G. | 2015 |
| Management of the low-flow head and neck vascular malformations in children: the sclerotherapy protocol | Leung, M. | 2014 |
| Dose-saving isolation procedure in percutaneous ethanol sclerotherapy for venous malformations | Kishi, K. | 2014 |
| Sclerotherapy of face and oral cavity low flow vascular malformations: our experience | Gorriz-Gomez, E. | 2014 |
| Haemangiomas and venous malformations of the head and neck: A retrospective analysis of endovascular management in 358 patients | Sachin, K. | 2013 |
| Vascular malformations of the head and neck | Kobayashi, K. | 2013 |
| Comparative analysis of intralesional sclerotherapy with sodium tetradecyl sulfate versus bleomycin in the management of low flow craniofacial soft tissue vascular lesions | Bajpai, H. | 2012 |
| Comparative analysis of intralesional sclerotherapy with sodium tetradecyl sulfate versus bleomycin in the management of low flow craniofacial soft tissue vascular lesions | Bajpai, H. | 2012 |
| Percutaneous treatment of facial venous malformations: a matched comparison of alcohol and bleomycin sclerotherapy | Spence, J. | 2011 |
| Percutaneous treatment of facial venous malformations: a matched comparison of alcohol and bleomycin sclerotherapy | Spence, J. | 2011 |
| Treatment of venous malformations with ethanolamine oleate: a descriptive study of 83 cases | Hoque, S. | 2011 |
| Digital subtraction angiography-guided percutaneous sclerotherapy of venous malformations with pingyangmycin and/or absolute ethanol in the maxillofacial region | Li, J. | 2011 |
| Management of palatal vascular malformation using absolute ethanol sclerotherapy | El-Hakim, I. | 2011 |
| Percutaneous sclerotherapy of massive venous malformations of the face and neck using fibrin glue combined with OK-432 and pingyangmycin | Chen, W. L. | 2010 |
| [Facial venous malformation presented with an unusual course] | Piza-Katzer, H. | 2009 |
| A pilot study on combination compartmentalisation and sclerotherapy for the treatment of massive venous malformations of the face and neck | Chen, W. L. | 2008 |
| Direct percutaneous ethanol instillation for treatment of venous malformation in the face and neck | Lee, C. H. | 2005 |
| Long-term results and quality of life after endovascular treatment of venous malformations in the face and neck | Rautio, R. | 2004 |
| Percutaneous sclerotherapy for venous malformations using polidocanol under fluoroscopy | Mimura, H. | 2004 |
| Advanced management of venous malformation with ethanol sclerotherapy: mid-term results | Lee, B. B. | 2003 |
| Sclerotherapy for congenital lesions in the head and neck | Kim, K. H. | 2004 |
| Experience of sclerotherapy and embolosclerotherapy using ethanolamine oleate for vascular malformations of the head and neck | Kaji, N. | 2009 |
| Color duplex-guided sclerotherapy for the treatment of venous malformations | Yamaki, T. | 2000 |
| Percutaneous ethanol sclerotherapy of venous malformations of the tongue | Johnson, P. L. | 2002 |
| Sclerotherapy to a large cervicofacial vascular malformation: a case report with 24 years' follow-up | Yildirim, I. | 2005 |
| Percutaneous sclerotherapy for lymphangioma and soft-tissue venous malformation located in the maxillofacial region in children and young adults | Graziuso, S. | 2006 |
| Ethanol sclerotherapy for the management of craniofacial venous malformations: the interim results | Lee, I. H. | 2009 |
| Intralesional sclerotherapy for subcutaneous venous malformations in children | Uehara, S. | 2009 |
| Masseteric venous malformations: Diagnosis, treatment, and outcomes | Rosbe, K. W. | 2010 |
| Percutaneous sclerotherapy for facial venous malformations: subjective clinical and objective MR imaging follow-up results | Spence, J. | 2010 |
| Sclerotherapy of voluminous venous malformation in head and neck with absolute ethanol under digital subtraction angiography guidance | Wang, Y. A. | 2010 |
| Direct Percutaneous Alcohol Sclerotherapy for Venous Malformations of Head and Neck Region without Fluoroscopic Guidance: Technical Consideration and Outcome | Baek, H. J. | 2011 |
| Low flow vascular malformation of the buccal mucosa treated conservatively by sclerotherapy (3% sodium tetradecyl sulfate) | Candamourty, R. | 2012 |
| Percutaneous sclerotherapy of vascular malformations in children using sodium tetradecyl sulphate: the Birmingham experience | Kok, K. | 2012 |
| Sclerotherapy for vascular malformations: complications and a review of techniques to avoid them | Odeyinde, S. O. | 2013 |
| Percutaneous sclerotherapy with ethanolamine oleate for venous malformations of the head and neck | Alexander, M. D. | 2014 |
| Digital subtraction angiography (DSA) guided sequential sclerotherapy for maxillofacial vein malformation | Meng, J. | 2014 |
| Ethanol sclerotherapy of head and neck venous malformations | Orlando, J. L. | 2014 |
| The use of sodium tetradecyl sulphate for the treatment of venous malformations of the head and neck | Alakailly, X. | 2015 |
| Efficacy of sclerotherapy with radio-opaque foam guided by digital subtraction angiography for the treatment of complex venous malformations of the head and neck | Chen, A. W. | 2015 |
| Nonsurgical management of vascular malformation of masseter | Jayaraman, V. | 2015 |
| Comparative outcomes for sclerotherapy of head and neck venous vascular malformation between alcohol and bleomycin | Songsaeng, D. | 2015 |
| Quantitative volumetric analysis of head and neck venous and lymphatic malformations to assess response to percutaneous sclerotherapy | Alexander, M. D. | 2016 |
| Foamed bleomycin sclerosis of airway venous malformations: The role of interspecialty collaboration | Azene, E. | 2016 |
| Direct intralesional ethanol sclerotherapy of extensive venous malformations with oropharyngeal involvement after a temporary tracheotomy in the head and neck: Initial results | Wang, D. | 2017 |
| Preliminary Experience With More Stable Polidocanol Foam in Sclerotherapy of Head and Neck Venous Malformations | Chen, A. W. | 2018 |
| Image guided sclerotherapy for the treatment of venous malformations | Gorman, J. | 2018 |
| Treatment of Head and Neck Venous Malformations with Sodium Tetradecyl Sulfate | Karimi, E. | 2018 |
| Craniofacial venous malformations treated by percutaneous sclerotherapy using polidocanol: a single-center experience | Grieb, D. | 2019 |
| Percutaneous sclerotherapy with gelified ethanol of low-flow vascular malformations of the head and neck region: preliminary results | Ierardi, A. M. | 2019 |
| Management of massive venous malformations by percutaneous injection of bleomycin combined with fibrin glue in the head and neck | Chu, H. | 2019 |
| Effect of foam and liquid bleomycin in the management of venous malformations in head and neck region: A comparative study | Helal, H. A. | 2020 |
| Percutaneous sclerotherapy with polidocanol under the guidance of ultrasound for venous malformations in children – A retrospective cohort study from a single tertiary medical center | Hou, F. | 2020 |

Complete list of 125 excluded Studies on full text assessment level
