# Supplementary material for: Interventional therapy of extracranial arteriovenous malformations of the head and neck—A systematic review
Source: PLoS One. 2022 Jul 15;17(7):e0268809. doi: 10.1371/journal.pone.0268809 (PMC9286278; doi:10.1371/journal.pone.0268809)
Supplement: Attachment — Submitted filename: Review PLOS ONE 19-05-21.docx [file pone.0268809.s009.docx]

*Review Notes*

*Overall*

Clear rationale and introduction of the void in our knowledge of AVMs. The writer is up to date in technical and anatomic jargon of the topic. Most tables and supporting data are complete and of value.

I do have major concerns in the methodological set up of the paper. Throughout the review different objectives are stated and it seems if the main goal was altered in the process of writing the manuscript. In addition various adapted risk of bias tools are available for non-RCT studies, these could be easily applied to the present review. Although the writers are critical and aware of the potential biases in the selected articles and opted to critically appraise in Table S2, I believe among others study design and sample size should be included (e.g. assessing the papers on directness of evidence and risk of bias). At this point the review lacks an easily assessable overview (i.e. flowchart or supporting figure) of the various treatments performed and its outcome in terms of for example success or complication rate, which I believe is the main objective of the review. Plenty of published reviews have coped with heterogeneity in results and alternatively analysed (e.g. semi-quantitative) and visualized these results in various supporting manners.

I would also strongly advise to re-write the paper with a native English speaker as the paper contains multiple errors which decreases the joy of reading.

*Abstract*

*Objective:* I believe the secondary aim is obsolete as a methodological assessment of the selected papers is one of the key elements of performing a systematic review.

*Methods:* “Criteria for meta-analysis were implemented.” I think you are meaning to say that a meta-analysis was performed?

*Results:* “Outcome analysis 38 varied with 45% of the authors using imaging parameters and 77% indicating the span of 39 follow-up of their entire patient collective” > this sentence is very vague for a new reader who was not involved in the research process, please re-write.

*Conclusion:* This conclusion is not clear from the results section, please align these paragraphs.

*Advances in knowledge:* Please add “data on AVMs /reporting standards addressing treatment of AVMs”, in this manner it seems you are referring to all data available.

*Introduction:*

Lines 69 -70: This objective is different of the objective in the abstract, even though outcome is a consequence of a treatment, you should define what is “most effective treatment” ? What outcome are you interested in?

Lines 70-71: See previous comment: evaluating the methodological quality of these papers is part of the systematic review.

Line 73: Why are you only addressing the papers from 2000? Perhaps due to interventional developments? Please add your rationale.

*Methods*

Lines 90-92: Consider to only mention your search in August 2020, it is OK to update your search once it gets outdated during the writing process, though only if it is performed in a similar fashion. I would recommend to add a second reviewer in the primary selection of eligible articles (or a substantial percentage of the eligible articles)

Lines 112-113: In this statement you are narrowing your research question and this should be used throughout your manuscript.

Line 123: To my opinion comparison in the PICO structure should be the other invention group you are comparing with, so that would be any primary treatment other than “sclerotherapy and embolization” or any patient in need of a secondary treatment and so on. At this point the paragraph after “Comparison” is just a brief sentence of your “Outcome” paragraph and does not give any additional information.

Line 142: The authors should seek to another approach to adequately assess directness of evidence and risk of bias in the selected articles. They could also consider to discuss the case reports separately from the other articles with a sample size of e.g. >15 or >20 patients.

*Results*
Line 252: “four articles reported either mean of maximum follow-up time only” > if this FU was longer than 1 year I guess these articles could still be included (as is stated in the methods). If so, please add why these papers were excluded.

For a meta-analysis, Pearson Chi square and Mann Whitney U tests are not appropriate.

*Discussion*

Another goal “representative meta-analysis” is added for the first time in the discussion part. The objectives should be similar throughout the manuscript.

The discussion shows that the authors do have substantial knowledge of the subject as they are able to discuss and reflect on the different treatment options. It would be of interest if the authors could add their view on what the suggested reporting standards should contain or focus on.

Nevertheless all information on the meta-analysis is hazardous to me, since I doubt if the authors have used the appropriate statistical approach.

Line 367-369: To my opinion a long list of Author et al is not reader-friendly, please re-write.

*Conclusion*

All recommendations for reporting standards should be placed in the discussion. In addition please explain why >99% (why not 90%?)a devascularisation is used and how this should be measured.

Also the strengths and limitations should be placed in the discussion.

*Fig 1 PRISMA flowchart*

- Please take note of the “simple” PRISMA set up of the flowchart in other published systematic reviews. This flowchart is quite chaotic and hard to follow for an interested reader.

*Supporting data:*

- Please add all supplemental data to one document to help the reader find the information they need to comprehend the message of your paper
- It would be better to give an summary score / rating to the separate articles in the table S2 in order to see which articles are the most appropriate to answer your research question. Also the table should stand on its own, meaning the table should be informative enough to fully understand independently of the paper. In my opinion the legend is too brief for a reader which is not as familiar with the subject as the writers.
- I understand the length of the Table S5 makes it unfavorable to add in the main body of the text, but as this table may be the most important finding of the study I would recommend the writers to think of another way to add this information to the main manuscript. Perhaps a clean flowchart with roughly categorized treatments and their success or complication rate would be of interest.
